# Phase I/II study of sequential therapy with irinotecan and S-1 for metastatic colorectal cancer

**DOI:** 10.1038/sj.bjc.6605432

**Published:** 2009-11-17

**Authors:** T Yoshioka, S Kato, M Gamoh, N Chiba, T Suzuki, N Sakayori, S Kato, H Shibata, H Shimodaira, K Otsuka, Y Kakudo, S Takahashi, C Ishioka

**Affiliations:** 1 Department of Clinical Oncology, Institute of Development, Aging and Cancer, Tohoku University, 4-1 Seiryo-machi, Aoba ward, Sendai 980-8575, Japan; 2 Department of Clinical Oncology, Faculty of Medicine, Yamagata University, 2-2-2 Iida-nishi, Yamagata 990-9585, Japan; 3 South Miyagi Medical Center, 38-1 Aza-nishi, Oogawara-machi, shibata-gun, Miyagi prefecture 989-1253, Japan; 4 Department of Molecular Immunology, Institute of Development, Aging and Cancer, Tohoku University, 4-1 Seiryo-machi, Aoba ward, Sendai 980-8575, Japan; 5 Sendai Medical Center, 2-8-8 Miyagino, Miyagino ward, Sendai 983-8520, Japan; 6 Miyagi Cancer Center, 47-1 Shiote-aza nodayama, Medeshima, Natori 981-1293, Japan; 7 Iwate Prefecture Central Hospital, 1-4-1 Ueda, Morioka 020-0066, Japan; 8 Department of Clinical Oncology, Akita University Faculty of Medicine, 1-1-1 Motomichi, Akita 010-8543, Japan

**Keywords:** irinotecan, S-1, metastatic colorectal cancer, sequential therapy

## Abstract

**Background:**

Both irinotecan (CPT-11) and S-1 are active against colorectal cancer; however, as S-1 is a prodrug of 5-fluorouracil (5-FU), 5-FU and its metabolites might inhibit the antitumour effect of CPT-11. Therefore, we designed a sequential combination, in which CPT-11 infusion was given on day 1 and S-1 was given orally at 80 mg m^−2^ per day on days 3–16 every 3 weeks.

**Methods:**

Twelve patients entered the phase I study, and the recommended doses were determined as a CPT-11 dose of 150 mg m^−2^ and an S-1 dose of 80 mg m^−2^.

**Results:**

In all, 36 patients entered the phase II study, of whom 4 and 16 had complete and partial responses. The overall response rate was 55.6% (95% confidence interval, 38.1–72.1%), and median progression-free survival was 7.7 months (95% confidence interval, 4.8–12.6 months). Grade 3 neutropenia was the most common haematological toxicity and occurred in 6.5% of 215 treatment courses. Grade 3 non-haematological toxicities included anorexia (1.4%) and diarrhoea (0.9%). There was no grade 4 toxicity of any kind.

**Conclusion:**

Our results suggest that this regimen is convenient, safe and promising, compared with conventional regimens for patients with metastatic colorectal cancer.

The development of new cytotoxic and molecular targeting agents has improved survival in patients with metastatic colorectal cancer (Douillard *et al*, 2000; Saltz *et al*, 2000; de Gramont *et al*, 2000; Cunningham *et al*, 2004; Hurwitz *et al*, 2004; Thirion *et al*, 2004). One of them is irinotecan (CPT-11), a potent inhibitor of topoisomerase I (Kawato *et al*, 1991; Pitot *et al*, 1997), which is combined with intravenous bolus 5-fluorouracil (5-FU) and leucovorin (LV) in the IFL regimen (Saltz *et al*, 2000) or with continuous intravenous infusion of 5-FU in the FOLFIRI regimen (Douillard *et al*, 2000). Two randomised phase III trials relating to IFL and FOLFIRI have shown survival benefits with a median overall survival time (MST) of 14.8–17.4 months (Douillard *et al*, 2000; Saltz *et al*, 2000). Recent reports have shown that FOLFIRI seems to be better tolerated (Benson and Goldberg, 2003), with less frequent toxicities such as diarrhoea, myelosuppression and infection than IFL; however, FOLFIRI needs an indwelling central venous catheter and a portable pump. Its usage is therefore complex and can cause problematic complications such as infection and thrombosis (Groeger *et al*, 1993; Verso and Agnelli, 2003).

S-1 is a new oral fluoropyrimidine developed by Taiho Pharmaceutical Co., Ltd (Tokyo, Japan), and is based on the biochemical modulation of 5-FU. This drug contains tegafur (FT), gimeracil (5-chloro-2,4-dihydoroxypyridine; CDHP) and oteracil (potassium oxonate; Oxo) at a molar ratio of 1 : 0.4 : 1 (Shirasaka *et al*, 1996a). FT, a prodrug of 5-FU, is absorbed well after oral ingestion and is converted to 5-FU mainly in the liver and tumour cells. CDHP, a strong inhibitor of dihydroxypyrimidine dehydrogenase, suppresses the degradation of 5-FU and maintains high 5-FU concentrations in the blood for long periods of time (Tatsumi *et al*, 1987; Shirasaka *et al*, 1996b). Oxo, which inhibits orotate pyrimidine phosporibosyl transferase, decreases the levels of 5-fluorouridine 5′-monophosphase and 5-FU incorporated into RNA only in the small intestine, and remains in the digestive tract after oral administration, reducing the gastrointestinal toxicity of 5-FU (Shirasaka *et al*, 1993).

Several phase II studies of S-1 monotherapy have shown that response rates ranged from 19 to 39%, and S-1 seemed to be a promising agent in patients with advanced colorectal cancer (Ohtsu *et al*, 2000; Van den Brande *et al*, 2003; Shirao *et al*, 2004). Combining CPT-11 with oral S-1 is compelling because the agents have different modes of action and home-based administration of oral S-1 is safe and convenient, compared with FOLFIRI. However, it has been shown that 5-FU or its metabolites may inhibit the activity of carboxylesterase, which converts CPT-11 to SN-38 (7-etyl-10-hydroxycamptothecin), a major active metabolite of CPT-11, from pharmacokinetic data (Sasaki *et al*, 1994; Falcone *et al*, 2001). If CPT-11 and S-1 are administrated simultaneously, S-1, a prodrug of 5FU, might inhibit the anti-tumour activity of CPT-11.

We therefore designed sequential therapy in which CPT-11 on day 1 and S-1 on days 3–16 were administered every 3 weeks, and conducted a phase I/II study of this regimen.

## Materials and methods

### Patients

The objectives of this study were to determine the maximum tolerated dose (MTD) and the recommended dose (RD), and to evaluate the toxicity and efficacy of the above sequential combination.

All patients had to have histologically confirmed colorectal cancer with measurable or evaluable lesions on the basis of the Response Evaluation Criteria in Solid Tumors Group (RECIST) criteria (Therasse *et al*, 2000). Previous chemotherapy regimen had to be one or less, but not including CPT-11 or S-1, and previous chemotherapy or radiotherapy had to have been completed at least 4 weeks before entry. Other eligibility criteria included an Eastern Clinical Oncology Group (ECOG) scale performance status of 2 or less; age between 20 and 75 years; the ability to ingest S-1 orally; life expectancy of at least 3 months; provision of written informed consent in accordance with government and institutional guidelines; adequate organ function defined by a WBC count from 4000 to 12 000 per mm^2^; an absolute neutrophil count of ⩾2000 per mm^2^; a haemoglobin value of ⩾9.0 g dl^−1^; a platelet count of ⩾10 × 10^4^ per mm^2^; AST and ALT levels within two times the normal upper limit or 150 IU dl^−1^ in the presence of liver metastasis; total bilirubin ⩽1.5 mg dl^−1^; and serum creatinine within the normal limits of each institute and/or creatinine clearance of ⩾50 ml min^−1^ by the calculation of Cockcraft–Gault. Exclusion criteria included active infection; diarrhoea (watery stools); severe pleural effusion or ascites; symptoms attributable to brain metastasis; serious complications such as intestinal paralysis, intestinal obstruction, interstitial pneumonia or pulmonary fibrosis; concomitant uncontrolled, non-malignant disease such as hypertension or cardiac, pulmonary, renal or hepatic disease; synchronous double cancer; a previous history of treatment for psychiatric diseases; pregnancy, possible pregnancy or lactation; a history of drug sensitivity to CPT-11 or S-1; flucytosin treatment; or judged ineligible for this protocol by the attending physician. The protocols were approved by the ethics committees of our institutions.

### Treatment regimens

CPT-11 was administrated as a 90-min intravenous infusion on day 1 and S-1 was administrated orally at 40 mg m^−2^ twice daily (i.e., 80 mg m^−2^ per day) within 1 h after breakfast and supper on days 3–16 every 3 weeks. The doses of S-1 were assigned on the basis of body surface area (BSA) as follows: BSA < 1.25 m^2^, 80 mg per day; 1.25 m^2^ ⩽ BSA < 1.5 m^2^, 100 mg per day; 1.5 m^2^ ⩽ BSA, 120 mg per day.

In the phase I study, the dose escalation of CPT-11 was conducted with a fixed S-1 dose of 80 mg m^−2^ per day. Four escalating dose levels of CPT-11 were prepared (Table 1). Level 1 was the starting dosage level but level 0 was also prepared, because level 1 might have been the MTD. The level 3 dose of CPT-11 was based on the upper limit recommended by the Japanese Government Health Care Insurance Organization. All toxicities were graded according to the Japanese version of the National Cancer Institute Common Toxicity Criteria (NCI-CTC v3.0) (JCOG and JSCO, 2004). DLT was defined as follows: (1) grade 4 leucocytopaenia; (2) grade 4 neutropaenia; (3) grade 3 febrile neutropaenia for 3 days or more; (4) grade 4 thrombocytopaenia or anaemia; (5) grade 3 or 4 non-haematological toxicity except alopaecia; (6) delay of more than 14 days in initiating the second cycle of therapy. To determine the MTD, only DLTs occurring during the first cycle of therapy were considered. At least three patients were entered at each level. If one patient at a certain dose level experienced DLT, then three additional patients were treated at the same dose level. Dose escalation was not allowed in individual patients. MTD was defined as the dose level that resulted in at least two of six patients developing DLTs. The RD of the phase II study was to be the dose immediately below the MTD. If MTD was not reached, the level 3 dosage was considered to be the RD.

In the phase II study, the dosage of CPT-11 was the RD, and eligibility and exclusion criteria were the same as those for the phase I study. The eligibility criteria of subsequent courses included the following: a WBC count of ⩾3000 per mm^2^; a neutrophil count of ⩾1500 per mm^2^; a platelet count of ⩾10 × 10^4^ per mm^2^; serum creatinine within the normal limits of each institute; total bilirubin ⩽1.5 mg dl^−1^; grade 1 or less non-haematological toxicity, except any alopaecia and pigmentation; no watery diarrhoea; and no infection causing fever of more than 38°C. If eligibility criteria were not met by day 35 of a given course, the patient was excluded from further study. If previous treatment courses were delayed or interrupted because of toxicity, the dose of CPT-11 was reduced to one level lower for subsequent courses, but the dosage of S-1 was not changed. If a further reduction of CPT-11 was needed, the patient was excluded from the study. If S-1 was interrupted within a course because of toxicity and the subsequent course could not be started within less than 14 days from the interruption of S-1, the patient was also excluded from this study. Once lowered, the dose of CPT-11 could not be increased.

Symptomatic treatment was given as required. A 5-hydroxytryptamine-3 receptor antagonist and dexamethasone were given to all patients in a 30-min infusion before the administration of CPT-11. The use of granulocyte colony-stimulating factors (GCS-F) was allowed if necessary. If possible, intestinal alkalisation by the intake of more than 1000 ml per day of alkaline water and/or 1.8 g per day of sodium bicarbonate and control of defecation with 2.0 g per day of magnesium oxide for 4 days after the administration of CPT-11 were performed to prevent delayed diarrhoea after CPT-11.

### Response evaluation and toxicity

Pretreatment evaluation included complete patient histories, physical examinations, complete blood cell counts, biochemistry involving liver and renal functions, urinalysis, tumour markers (CEA, CA19–9, etc.), chest roentgenogram, electrocardiogram and computed tomographic scans of the abdomen and chest. According to NCI-CTC version 3.0 (JCOG and JSCO, 2004), toxicity and laboratory variables in complete blood cell counts, biochemistry and urinalysis were assessed weekly during the first course, on days 1 and 15 from the second through to the sixth course and at least once during subsequent courses. CT scans were repeated to evaluate lesions every two courses, and tumour markers were measured at the same time. Responses were evaluated according to the RECIST criteria (Therasse *et al*, 2000). Complete and partial responses required subsequent confirmation of response after an interval of at least 4 weeks.

### Statistical considerations

The phase II study was designed to test the null hypothesis that the true response probability is not less than the clinically significant level of 25%. The response rate of first-line FOLFIRI was 50%. The response rate of this study was expected to be 45%, because it was probable that patients had previously received some regimens of chemotherapy. The probability of accepting treatment with a response probability (25%) was *P*=0.05. The probability of rejecting treatment with a response rate (45%) was *P*=0.2; therefore, the required number of patients was estimated to be 32. We planned to evaluate toxicity and efficacy after enroling 16 patients. Had this regimen been toxic or had there been fewer than four patients with a response, this study would have been halted. In the phase II study, survival was calculated from treatment initiation data by the Kaplan–Meier method.

## Results

### Patient characteristics

We enroled 12 patients in the phase I study between March 2005 and January 2006 (Table 2). All 12 patients were evaluated for toxicity and had evaluable lesions for response. The median age of patients was 55.5 years (range, 37–71), nine had an ECOG PS of 0 and three had an ECOG PS of 1. The primary lesions were in the colon in seven patients and in the rectum in five patients, and 10 primary lesions were resected. The metastatic sites were the lung in two patients, liver in six, lymph nodes in nine and peritoneum in one. Nine patients had received chemotherapy earlier with bolus 5-FU and LV regimen, and three patients were chemotherapy naive.

In the phase II study, all 36 patients enroled between February 2005 and February 2008 met all eligibility requirements and received at least one course of treatment. Patient characteristics are summarised in Table 2, and all patients were evaluated for toxicity and response. The median age of patients was 58.5 years (range, 21–73 years); 30 patients had an ECOG PS of 0 and 6 patients had an ECOG PS of 1. Primary sites were the colon in 21 patients and the rectum in 15 patients, and 9 primary lesions were resected. Metastatic sites were the lung in 11 patients, liver in 20, lymph nodes in 12, intrapelvic cavity in 8, peritoneum in 5, pleura in 1, ovary in 2 and spleen in 1. Five patients had received chemotherapy earlier with bolus 5-FU and LV regimen, and 31 patients were chemotherapy naive.

### Determination of MTD in the phase I study

In the phase I study, all patients were evaluable for adverse reactions and completed one or more cycles of treatment. At level 2, one patient exhibited grade 3 ileus on day 20 of the first cycle, but no other DLT occurred in phase I. The MTD was not reached, and we determined that the level 3 dosage was the RD, which was a CPT-11 dose of 150 mg m^−2^ with an S-1 dose of 80 mg m^−2^. Adverse reactions in the phase I study are summarised in Table 3.

### Safety

All 36 patients enroled in the phase II study were assessable for safety, and received 215 treatment courses (median, 5 courses; range, 1–20 courses). In these 36 patients and 215 courses, treatment delay within 2 weeks, dose reduction or both occurred in 8, 3 and 2 patients and 45, 5 and 2 courses, respectively. All treatment delays were due to delays in recovery of neutropaenia, and dose reductions were due to grade 3 anorexia and general fatigue. Four patients refused to continue treatment after one course because of obstruction of the gastrointestinal tract, skin rash, anorexia or stomatitis.

The overall incidences (%) of haematological and non-haematological toxicities in the phase II study are listed in Table 4. Grade 3 neutropaenia was the most common adverse event and occurred in 6.5% of the 215 treatment courses, but GCS-F was not used. No patient had febrile neutropaenia. Major non-haematological toxicities were liver dysfunction, anorexia, stomatitis, diarrhoea and alopecia. Grade 3 non-haematological toxicities were anorexia (1.4%), diarrhoea (0.9%), nausea (0.5%), vomiting (0.5%), ileus (0.5%) and general fatigue (0.5%). There were no serious unexpected adverse events and no treatment-related deaths.

### Efficacy

In the phase I study, all patients had lesions that could be evaluable for response, but one patient was not evaluable (NE) because the protocol was discontinued due to DLT. Patients included one with CR, four with PR, four with SD and two with PD, yielding a response rate of 41.7% (Table 5).

**Figure 1:**
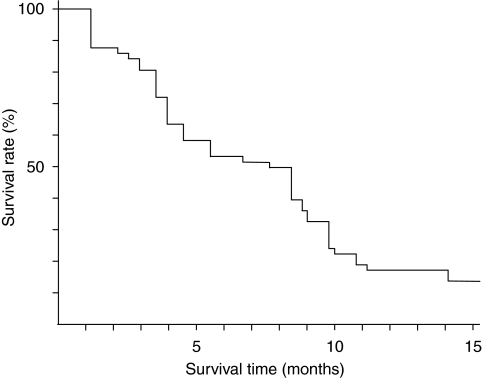
Progression-free survival time of 36 patients with metastatic colorectal cancer who received sequential therapy with irinotecan and S-1 in the phase II study. The median progression-free survival was 7.7 months (95% confidence interval, 4.8–12.6 months).

In the phase II study, all 36 patients had at least one measurable lesion. Response data are shown in Table 5. The overall response rate was 55.6% (95% confidence interval, 38.1–72.1%); 4 patients had CR, 16 had PR, 6 had SD, 6 had PD and 4 were defined as NE. Two of four patients with CR had had lymph node metastases, one had had liver metastases and one had had intrapelvic masses. Subgroup analysis based on previous chemotherapy showed that the response rate was 58.1% (18 out of 31) among those who had not undergone chemotherapy earlier and 40% (two out of five) among those who had undergone chemotherapy earlier. At a median follow-up time of 12 months, the median progression-free survival (PFS) time was 7.7 months (range, 1.2–19.9+months; 95% confidential interval, 4.8–12.6 months) (Figure 1). As survival times in more than half of the patients were not yet evaluable, the MST could not be calculated.

## Discussion

This study determined the MTD and RD, and also evaluated the toxicity and efficacy of the sequential combination of CPT-11 and S-1 in patients with metastatic colorectal cancer. As there was only one DLT, and the RD was 150 mg m^−2^ and 80 mg m^−2^ of CPT-11 and S-1, respectively, MTD could not be determined. Our phase II results showed that CPT-11 and S-1 were very effective, with a response rate of 55.6% and a median PFS of 7.2 months. These results are comparable with those of FOLFIRI (Douillard *et al*, 2000), and this combination might therefore be considered as a substitute of FOLFIRI, especially because of the lower toxicity.

Toxicity was very mild and the number of adverse events was very small. There were no grade 4 adverse events, and although the most common grade 3 adverse effect was neutropaenia, its incidence was only 6.5%. GCS-F did not have to be used and no febrile neutropaenia occurred. No severe non-haematological toxicities occurred, and the incidence of grade 3 non-haematological toxicities was also very low. This shows that our regimen is very safe and manageable on an outpatient basis.

Goto *et al* (2006) reported another regimen of CPT-11 combined with S-1 in patients with advanced colorectal cancer. In their regimen, CPT-11 was given on day 1 and S-1 on days 1–14, repeated every 3 weeks. Their response rate was 62.5% and the median PFS was 8.0 months; however, all patients in their study were chemotherapy naive, whereas 5 of 36 pretreated patients were involved in our study. As subgroup analysis on the basis of earlier chemotherapy showed that the response rate was 58.1% (18 of 31) among patients who had not undergone previous chemotherapy in our study, it is difficult to make a simple comparison between their study and ours. In terms of the overall number of all grades of adverse effects, their regimen seemed to have more adverse effects than ours, but figures for the numbers of grade 3 or more were similar in both regimens. As the two regimens could not be fully compared, as there was no randomised trial, we could not identify the superior regimen.

Capecitabine is another oral fluoropyrimidine derivative. Patt *et al* (2007) conducted a phase II study of capecitabine plus 3-weekly irinotecan (XELIRI regimen) as first-line chemotherapy for metastatic colorectal cancer. Their response rate was 50% and median PFS was 7.8 months; however, the incidence and degree of adverse events were high: grade 3/4 neutropaenia (25%), diarrhoea (20%), vomiting (16%), dehydration (10%), nausea (6%), abdominal pain (6%) and hand–foot syndrome (6%); most patients (94%) required a dose reduction of either CPT-11 or capecitabine or both. In our study, the toxicity profile was lower, with treatment delay and/or dose reduction occurring in only 25% of all treatment courses, and suggesting that S-1 is superior to capecitabine as an oral partner of CPT-11.

Oral uracil/tegafur (UFT) with oral LV was also combined with CPT-11. Mackay *et al* (2003) conducted a phase I/II trial of UFT, LV and CPT-11 inpatients with advanced colorectal cancer. Their response rate was 19%, whereas 35% of patients at the RD level needed dose reductions because of grade 3/4 neutropaenia and grade 3 diarrhoea. Taken together, these results suggest that CPT-11 and S-1 are safer and more active than UFT, LV and CPT-11 in patients with metastatic colorectal cancer.

The incidence of diarrhoea was very low in our study, with grade 3 diarrhoea occurring in only 0.9% of all courses. CPT-11 is hydrolysed by hepatic carboxylesterase to create 7-ethyl-10-hydroxy-camptothecin (SN-38), and a portion of SN-38 undergoes subsequent conjugation by UDP-glucuronyltransferase to form inactive SN-38-glucuronide (SN38-Glu) in the liver. CPT-11, SN-38 and SN38-Glu are mainly excreted into bile and discharged into stool, but some amount is reabsorbed into the enterohepatic circulation to a certain extent by intestinal cells. Enterocolitis caused by high levels of SN-38 and/or CPT-11 retained for long periods of time in the small intestine is thought to be the direct cause of diarrhoea associated with CPT-11 (Ikegami *et al*, 2002; Alimonti *et al*, 2004). It is thought that oral alkalisation combined with control of defecation could prevent CPT-11-induced diarrhoea (Takeda *et al*, 2001; Alimonti *et al*, 2004). In our study, oral alkalisation and control of defecation were performed as much as possible, and might be the reason for the low incidence of diarrhoea.

In conclusion, our results showed that the RD of CPT-11 and S-1 in our regimen was 150 mg m^−2^ and 80 mg m^−2^, respectively, and sequential therapy with CPT-11 and S-1 is safe and effective compared with conventional regimens in patients with advanced colorectal cancer. This promising regimen might be an alternative to FOLFIRI. A randomised control study comparing FOLFILI and our protocol regimen with or without molecular targeting agents is warranted.

## Figures and Tables

**Table 1:** Phase I dose escalation

**Dosage level**	**CPT-11 (mg m^−2^)**	**TS-1 (mg m^−2^)**	**No. of enroled patients**
0	75	80	0
1	100	80	3
2	125	80	6
3	150	80	3

**Table 2:** Patient characteristics

	**Phase I (*n*=12)**	**Phase II (*n*=36)**
Median age, years (range)	55.5 (37–71)	58.5 (21–73)
Male/female	9/3	16/20
		
*ECOG performance score*		
0	9	30
1	3	6
		
*Primary lesions*		
Colon	7	21
Rectum	5	15
		
*Histological differentiation*		
Well	5	9
Moderately	5	20
Poor	2	7
		
*Primary site*		
Yes	2	9
No	10	27
		
*Sites of metastasis*		
Liver	6	20
Lung	2	11
Lymph node	9	12
Peritoneum	1	5
Pelvic cavity	0	8
Ovary	0	2
Pleura	0	1
Spleen	0	1
		
*Prior chemotherapy*		
Yes	9	6
No	3	30

Abbreviation: ECOG=Eastern Clinical Oncology Group.

**Table 3:** Adverse reactions during the first cycle in phase I

**Dosage level**	**1 (*n*=3)**				**2 (*n*=6)**				**3 (*n*=3)**			
**Grade of toxicity**	**1**	**2**	**3**	**4**	**1**	**2**	**3**	**4**	**1**	**2**	**3**	**4**
*Haematological*												
Leucopaenia	0	0	0	0	1	1	0	0	1	0	0	0
Neutropaenia	2	0	0	0	0	1	0	0	0	0	0	0
Anaemia	0	1	0	0	1	0	0	0	0	0	0	0
												
*Non-haematological*												
Anorexia	1	2	0	0	0	2	0	0	0	0	0	0
Nausea	1	0	0	0	4	1	0	0	1	0	0	0
Diarrhoea	1	0	0	0	1	1	0	0	2	0	0	0
Stomatitis	0	0	0	0	0	0	0	0	1	0	0	0
Constipation	1	1	0	0	0	0	0	0	0	0	0	0
Ileus	0	0	0	0	0	0	1	0	0	0	0	0
Fever	1	0	0	0	1	0	0	0	0	0	0	0
General fatigue	0	1	0	0	1	0	0	0	0	0	0	0
Rash	0	0	0	0	0	1	0	0	0	0	0	0
Alopecia	2	0	0	0	4	0	0	0	1	0	0	0
DLT	0				1				0			

Abbreviation: DLT=dose limiting toxicity.

**Table 4:** Adverse reactions in all phase II 215 treatment courses

	**Grade of toxicity**					
**Toxicity (*n*=215)**	**1**	**2**	**3**	**4**	**All grades (%)**	**Grades 3 and 4 (%)**
*Haematological*						
Leucopaenia	5	41	3	0	22.8	1.4
Neutropaenia	11	50	14	0	34.9	6.5
Anaemia	15	29	2	0	21.4	0.9
						
*Non-haematological*						
Anorexia	12	14	3	0	13.5	1.4
Nausea	11	8	1	0	9.3	0.5
Vomiting	13	4	1	0	8.4	0.5
Diarrhoea	18	20	2	0	18.6	0.9
Stomatitis	19	3	0	0	10.2	0
Constipation	1	0	0	0	0.5	0
Ileus	0	0	1	0	0.5	0.5
Fever	1	0	0	0	0.5	0
General fatigue	6	6	1	0	6.1	0.5
Rash	0	3	0	0	1.4	0
Tearing	3	3	0	0	2.8	0
Alopecia	139	0	0	0	64.7	0
Bilirubin	20	4	0	0	11.2	0
GOT	40	1	0	0	19.1	0
GPT	35	3	0	0	17.7	0

**Table 5:** Overall response

	**No. of patients**	**CR**	**PR**	**SD**	**PD**	**NE**	**Response rate (%)**
*Phase I*							
Overall	12	1	4	4	2	1	41.7
							
*Level*							
1	3	0	1	1	1	0	33.3
2	6	0	2	2	1	1	33.3
3	3	1	1	1	0	0	66.7
							
*Phase II*							
Overall	36	4	16	6	6	4	55.6
							
*Prior chemotherapy*							
No	31	4	14	5	4	4	58.1
YES	5	0	2	1	2	0	40

Abbreviation: NE=not evaluable.
